# The caretaker-reversible Tarsorrhaphy

**DOI:** 10.1007/s10792-024-03310-7

**Published:** 2024-10-01

**Authors:** Jonathan E. Lu, Tiffany Ho, Desmond Chin, Christine Ryu, Sandy Zhang-Nunes

**Affiliations:** Keck School of Medicine of the University of Southern California, USC Roski Eye Institute, San Pablo Street 1450, Los Angeles, CA 90033 USA

**Keywords:** Tarsorrhaphy, Cornea, Oculoplastics, Oculoplastic surgery

## Abstract

**Purpose:**

To present a modification of the reversible tarsorrhaphy that can be opened and reclosed as necessary by caretakers and the patient.

**Methods:**

Retrospective case series of patients who underwent the reversible tarsorrhaphy.

Materials included intravenous (IV) tubing as bolsters and 4–0 polypropylene suture. The 4–0 suture is first passed through and through one end of IV tubing approximately 20 mm in length. Starting on the lateral upper lid and approximately 4 mm above the lash line, the suture is placed through the skin and into the tarsus. The suture exits through the eyelid gray line. These steps through the eyelid are repeated in the opposite direction. An air knot is tied above the upper eyelid near the base of IV tubing. A second air knot can be tied higher to provide a handle easily allowing the caretaker to close the eyelid.

**Results:**

Included were 13 patients (ages 21–95-yeas), indications included lagophthalmos secondary to cicatricial changes from burns (2), keratouveitis (1), neurogenic palsy (3), neurotrophic ulcer (6), and cicatricial changes from skin cancer (1). There were no reported difficulties in maintaining the tarsorrhaphy by either family members or healthcare providers. The first tarsorrhaphy for each patient lasted between 3–19 weeks, with an average of 8 weeks. All were well tolerated.

**Conclusions:**

The caretaker-reversible tarsorrhaphy can be used as a temporizing measure. The technique balances the need for ocular protection with the need for examination/treatment by health care professionals and, equally importantly, the patients and caretakers.

## Introduction

Tarsorrhaphy has been an effective technique for corneal protection and healing [1]. A wide range of approaches has been used, ranging from the less invasive such as tape, cyanoacrylate [2] and botulinum toxin A injections into the levator muscle [3]. However, tarsorrhaphy with adhesive tape require frequent maintenance and risk of corneal abrasion, glue tarsorrhaphies risk damage to ocular surface and are not reversible for ocular exam, botulinum toxin can infiltrate to rectus muscles and does not allow for reversal during the weeks-months that the toxin is effective. [4] Surgical techniques range from temporary or permanent techniques. Suture techniques with bolsters and non-absorbable sutures through the gray line or tarsus have been described [5–9].

Here we describe a modification to the traditional tarsorrhaphy that allows not only medical personnel, but also the patient and their caretakers to open and close the tarsorrhaphy. We also present the largest case series to date undergoing a truly reversible tarsorrhaphy. Within our own institution, the caretaker-reversible tarsorrhaphy can be performed in various settings and utilizes cost-effective, widely available materials.

## Methods

We conducted a retrospective review of the medical records al all patients who underwent our modified temporary tarsorrhaphy during a 6-year period (2014–2020) at two academic institutions. Exclusion criteria included follow-up of less than 2 weeks of the tarsorrhaphy, and concurrent periocular surgeries. This study was exempt from institutional review board given retrospective and de-identified nature of this study.

**Fig. 1:**
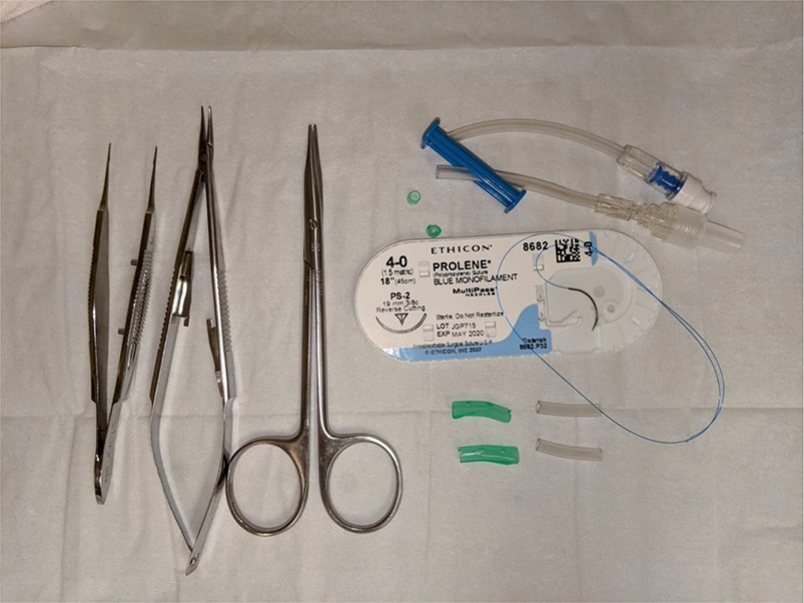
Materials for procedure

**Fig. 2:**
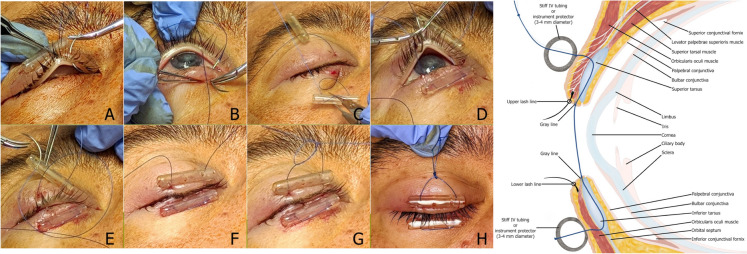
Reversible tarsorrhaphy illustrated. Suture should exit through the eyelid grayline (Figure 2, A) then into the grayline of the opposite eyelid in a parallel position (Figure 2, B), then passed through and through the cross section of the second IV tubing bolster (Figure 2, C). These steps are repeated for the second arm of the suture near the medial aspect of the eyelid (Figure 2, D,E, F). A sliding knot is tied above the upper eyelid near base of IV tubing (Figure 2, G, H) resulting in a completed surgery (Figure 2, I)

**Fig. 3:**
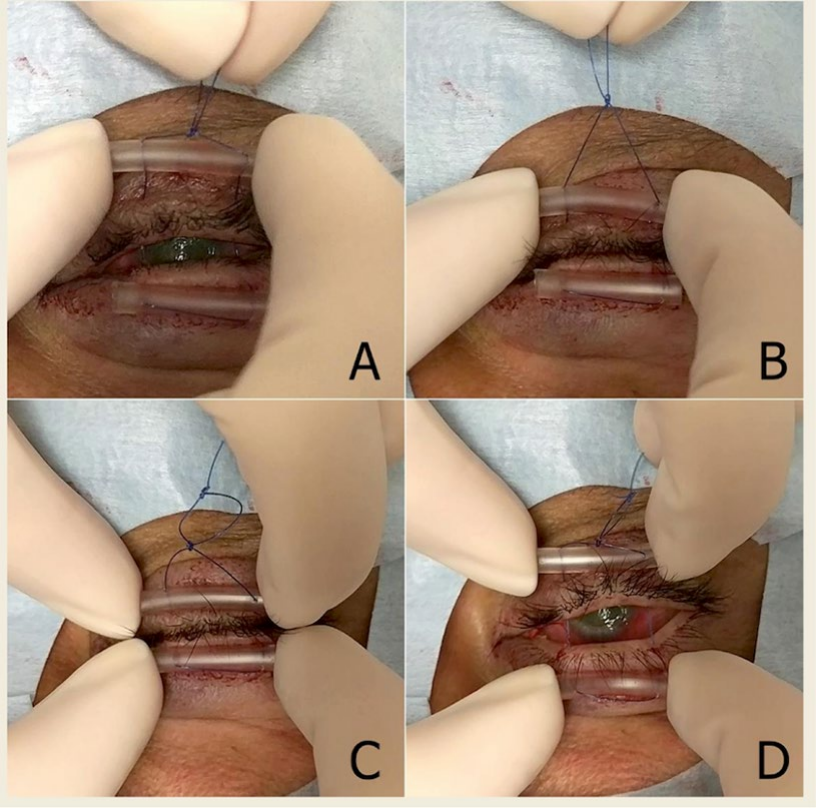
Demonstration of the tarsorrhaphy opened and closed

*The surgical technique:* In either the operating room or clinic, the patient is placed in the supine or semi-sitting position. The upper and lower eye lid margins are first anesthetized in subcutaneous fashion with 1% lidocaine with epinephrine; 1:100,000 using a 30-gauge needle. For bolster material, two 2-cm strips of soft intravenous (IV) tubing are used to protect eyelid from the cheese-wiring effect of suture on skin (Fig. 1). A double armed 4–0 prolene suture with a cutting needle is first passed through and through the cross section of the IV tubing 2 mm from the cut end. As described in a previous study by McInnes et al. [10], 4–0 suture is not only stronger and cheese-wiring is less common when compared to smaller 5–0 or 6–0 sutures. Starting on the lateral lower lid and 5 mm below the lash line, the first suture is placed through the skin and into tarsus muscle (Fig. 2). The suture should exit through the eyelid grayline (Fig. 2A) with surgeon flipping the eyelid to ensure that bite is not full thickness. The needle should then proceed to enter the grayline of the opposite eyelid in a parallel position (Fig. 2B) with a strong bite of tarsus and exit through the skin below the lash line. The needle is then passed through and through the cross section of the second IV tubing bolster (Fig. 2C). These steps are repeated for the second arm of the suture near the medial aspect of the eyelid (Fig. 2D,E, F). An air-sliding knot is tied above the upper eyelid near base of IV tubing (Fig. 2G, H) resulting in a completed surgery (Fig. 2I). The knot can slide to the base of the IV tubing to cinch the eyelids close (Fig. 3A and B), or slid in reverse to allow the eyelids to open (Fig. 3, C and D).

## Results

Thirteen patients were included in the study. The median age was 59 years old (range 21–95 years old). Retrospective case series: 13 patients (ages 21–95-yeas) received temporary tarsorrhaphies. Indications for surgery included lagophthalmos secondary to cicatricial changes from burns (2), keratouveitis (1), neurogenic palsy (3), neurotrophic ulcer (6), and cicatricial changes from skin cancer (1).

The duration of first temporary tarsorrhaphy ranged from 3–19 weeks (average duration 8 weeks). The intended replacement schedule was approximately 4–8 weeks. Functioning tarsorrhaphies were removed ahead of the schedule in cases of definitive reconstructive surgery (3 cases), healed ocular surface (4), or permanent temporary tarsorrhaphy (2). There were two cases of spontaneous extrusion.

Three patients required more than 1 temporary tarsorrhaphy (range 2–4 repetitions). These patients included 2 patients who had large basal cell carcinoma excisions, one of which eventually preferred to undergo permanent tarsorrhaphy, and the other patient had multiple repetitions due to awaiting anesthesia clearance for definitive eyelid reconstructive surgery. The third patient requiring repeat tarsorrhaphies was subsequently managed with topical therapy and bandage contact lenses.

There were no events of corneal decompensation or loss of eye; there were no procedure related complications.

## Discussion

The ideal tarsorrhaphy for corneal protection is one that meaningfully protects the cornea, is cost and time efficient, low maintenance, without significant adverse effect profile, and can be readily reversed. There are numerous approaches that have achieved various combinations of these traits. However, historically the reversible techniques often had a tradeoff of ease versus effective corneal coverage (such as with tape), or are reversible only in a relative sense (such as suture tarsorrhaphies).

The proposed technique in this study is a variation of similar dynamically reversible tarsorrhaphies. These include the drawstring temporary tarsorrhaphy [5] or the zipper tarsorrhaphy [11]. In our technique, the use of only two bolsters is a faster and simpler technique compared to the drawstring tarsorrhaphy. It possible to eliminate the need for the 3rd bolster due to passing the suture through and through the IV tubing resulting in more friction and thus more control in the final result. Additionally our use of IV tubing compared is readily available in the clinic setting, compared to some techniques such as the zipper tarsorrhaphy^10^ that require rubber urinary catheters.

Reversibility is an important feature for regular exams and treatment by physicians, eye drop application by patients and family members, and allowing the patient breaks from total occlusion of the eye, all this while offering corneal protection. In our study population, this was most pertinent to patients with keratouveitis for whom eyelid protection of the cornea alone was not sufficient, as they also required active topical therapy for inflammation. Additionally reversibility can be valuable in the intensive care unit setting or elderly patients in which hospitalization induced delirium and fatigue from disrupted circadian rhythms is a risk.

Patients and their caregivers demonstrated capacity and comfort in the clinic with opening and closing our caregiver tarsorrhaphy. Future directions include comparative study of ophthalmic outcomes, as well as subjective measures of patient/caregiver comfort and satisfaction with the reversibility feature.

Overall our proposed variation of the reversible tarsorrhaphy was safe, efficient, had good longevity, and appeared effective in corneal protection.

## Meeting presentation

American Society of Ophthalmic Plastic and Reconstructive Surgery, Fall Meeting 2019, San Francisco CA. October 2019.

## Data Availability

No datasets were generated or analysed during the current study.
